# Ovarian Oxidative Stress Induced Follicle Depletion After Zona Pellucida 3 Vaccination Is Associated With Subfertility in BALB/c Mice

**DOI:** 10.3389/fvets.2022.814827

**Published:** 2022-02-18

**Authors:** Beibei Zhang, Guanggang Qu, Yuchen Nan, En-Min Zhou

**Affiliations:** ^1^Department of Preventive Veterinary Medicine, College of Veterinary Medicine, Northwest A&F University, Yangling, China; ^2^Scientific Observing and Experimental Station of Veterinary Pharmacology and Diagnostic Technology, Ministry of Agriculture, Yangling, China; ^3^Shandong Binzhou Animal Science and Veterinary Medicine Academy, Binzhou, China

**Keywords:** zona pellucida 3, subfertility, ovary, follicle depletion, oxidative stress, apoptosis

## Abstract

Impaired follicular development associated with autoimmune ovarian disease (AOD), is a typical side effect of ZP3 vaccine-induced subfertility and contributes to the fertility decline, but the mechanism is unknown. In this study, a AOD model was established with recombinant mouse zona pellucida 3 (mZP3) protein in the BALB/c mice, and co-administrated with 0.5 mg/kg antioxidant stress drug sodium selenite (SS), whereas intraperitoneal injection was used and the relationships among oxidant stress (OS), follicle loss and fertility were evaluated. Here we demonstrated that ZP3 vaccination elicited high antibody titers and correlated with reductions of ovarian follicle numbers in both fertile and infertile mice, whereby magnitudes of both factors were negatively correlated with litter size. Moreover, increased OS in ovaries of mZP3-immunized mice was related to high levels of reactive oxygen species (ROS) and malondialdehyde (MDA), and is accompanied by a decrease in the total antioxidant capacity (TAC) of ovaries. Meanwhile, activation of caspase-3 and caspase-9 along with increased Bax and decreased Bcl-2 levels were observed, indicating the ongoing apoptosis of ovarian cells. Notably, inhibition of OS with SS reduced ovarian ROS and apoptosis levels, which was consisted with restoration of follicle numbers. More importantly, SS treatment when co-administered concurrently with mZP3 immunization led to significantly improved fertility (*P* < 0.05) and the average litter size of the mZP3-vaccinated SS-treated group increased by ~29.2% as compared with that of the vaccinated but untreated group. In conclusion, infertility caused by ZP3 vaccination was mechanistically associated with ovarian OS which triggered depletion of ovarian follicles.

## Introduction

Zona pellucida 3 (ZP3), the primary oocyte receptor for sperm recognition, has been recognized as a potential candidate for use as birth controlling target in mammalian species by immunizing using ZP3 vaccination (1–3). However, ZP3 immunization is associated with AOD and typically induces symptoms of premature ovarian failure (POF) (4, 5), thus greatly limiting its applicability as a contraceptive (5). Interestingly, development of ovarian pathology could be blocked after disruption of the ZP3 pathogenic epitopes (6) or improved after adoptive transfers of CD4^+^CD25^+^ Tregs (4, 7). Although individual differences are observed among experimental subjects, ZP3-induced ovarian inflammation is closely associated with ovarian dysfunction and fertility, with severe inflammation often accompanied with fewer follicles and smaller litter sizes (4, 8). Of note, vaccination-induced ovarian dysfunction is characterized by follicular atresia, vacuolation, luteinization and other signs that may be responsible for the reduction or total elimination of follicles and subsequent infertility (9, 10). Therefore, ovarian inflammation may be related to these pathological effects despite its role in fertility decline remains unclear.

Reactive oxygen species (ROS), a by-product of mitochondrial processes associated with energy metabolism, participates in the regulation of follicle maturation during different developmental stages when presents at physiological concentrations (11). However, abnormal persistence of ROS in tissues, especially during chronic inflammation, is viewed as a key factor associated with inflammation-related diseases (12). At the site of inflammation, ROS-induced oxidant stress (OS) is involved in irreversible injury of cells or tissues through DNA damage or peroxidation (13), causing pathological changes which could adversely impact the reproductive system, nervous system, respiratory system, etc. (14–16). Regarding to reproductive diseases, it has been confirmed that ROS is excessively accumulated in ovarian tissues during inflammation-mediated ovarian diseases and contributes to disease progression (17, 18). Meanwhile, there was report suggested that ovarian inflammation triggered by pathogenic T cells specific recognizing certain ZP3 epitopes maybe accompanied by ROS accumulation (19). Nevertheless, this hypothesis has not yet been evaluated in fertility studies and the link between OS and ovarian pathology remains elusive.

Selenium (Se) is considered as an essential trace mineral, which plays an important role in the health of animals and humans at the cell and organism levels (20). The biological functions of Se are mainly performed by selenoproteins, and related functions are conserved among mammal species (21, 22). As the main component of selenoprotein, Se can exert its catalytic and anti-oxidant functions by stabilizing structure and enzymatic activities, as well as regulate physiological processes such as DNA synthesis, clearance of harmful peroxides and redox signal control (20, 23), whereas suitable low-concentration Se can promotes activation of selenoproteins and suppress ROS production by trapping electrons released during oxidative phosphorylation as an anti-oxidative effect (24). Inorganic (selenates and selenites) and organic (selenomethionine and selenocysteine) Se are two forms of Se in the dietary profile for supplementation though oral and injection (25, 26). Generally, organic Se is preferred due to higher absorption efficiency and metabolic stability whereas the blood Se concentration can be boosted quickly after supplementation, leading to the accumulation in the systemic circulation. However, chronic Se poisoning occurs after long-term administration (26, 27). Conversely, although inorganic Se demonstrates lower utilization efficiency after administration, the toxicity of inorganic Se requires high-dose drug or high local concentration (26, 28), which makes inorganic Se as favorable and injectable supplements for applying in the critical periods in the production cycle (28), or cancer therapeutics (29, 30).

Sodium selenite (SS) is the representative form of inorganic Se and there are conclusive evidences suggested that SS can prevent ROS accumulation in ovaries and prevent or reverse OS-induced ovarian dysfunction (31). The level of hormones and development of follicles were significantly improved after inhibition of ROS production using SS, whereas the capabilities of total antioxidant capacity (TAC) was also enhanced (32). More importantly, the similar phenomenon were observed from randomized double-blind placebo-controlled trials in pregnant women, and the SS-induced improvement of antioxidant capacity has been shown to be closely related to fertility (33, 34). Therefore, the connection among fertility, OS and follicle during the ZP3-administration period has investigated in present study. Here, the mZP3 protein vaccination was used to establish an AOD mouse model. The OS level, follicle number and apoptosis level were evaluated performed after periodic mZP3 immunizations to verify the contribution of OS to follicle depletion of ZP3-induced subfertility mice with or without concomitant sodium selenite (SS) treatment, and ultimately to reveal the relationship between OS and fertility.

## Materials and Methods

### Animals and Antigens

Adult female BALB/c mice (6–8 weeks old) were purchased from Dashuo Biotech (Chengdu, Sichuan, China) received sterile water and food under daily 12-h light/12-h dark cycle conditions. All animal experiments in this study were approved by the Committee on Ethical Use of Animals of Northwest A&F University. Care of animals was conducted with strict adherence to the guidelines of Northwest A&F University Institutional Committee and every effort was made to minimize animal suffering.

Recombinant mouse ZP3 protein fused to glutathione S-transferase (GST) tag was produced by using a constructed plasmid pGEX-4T-1-mZP3 with the prokaryotic expression system as previously reported (35). The control protein GST was obtained using the aforementioned backbone plasmid and expression system as well. Briefly, pGEX-4T-1 was transformed into BL21 (DE3) cells (TransGen Biotech, Beijing, China) then protein expression was induced by incubation of cells (after the OD_600nm_ value of bacteria reached 0.6–0.8) with 0.2 M isopropyl-β-D-thiogalactoside (IPTG) at 28°C for 8 hours shaking culture (180 rpm). Next, a supernatant containing GST protein was obtained after ultra-sonication of bacterial followed by a high-speed centrifugation (12,000 × *g*) at 4°C. Purification of GST protein from the supernatant was conducted according to the manufacturer's instructions using BeyoGold™ GST-tag Purification Resin (Beyotime Biotechnology, Beijing, China). Protein concentrations were quantified using a BCA protein assay kit (Thermo Fisher Scientific; Waltham, Massachusetts, USA).

### Animal Immunization and SS Treatment

Seventy-five female BALB/c mice were permitted to acclimate to their new environment for 7 days without any treatment. Referring to the previous study (36), after acclimation the mice were administered by intramuscular injections (0.1 mg/kg) of estradiol benzoate (EB; Sigma-Aldrich, St. Louis, MO, USA) for 3 consecutive days to synchronize their estrus cycles, and the signs of a large number of keratinized epithelial cells and a small number of nucleated epithelial cells in the vaginal wash are defined as estrus period though smear observation. Next, mice were randomly divided into three groups (*n* = 25): (a) mZP3+SS; (b) mZP3+Saline; and (c) GST+Saline (negative control group). For mice receiving the immunogen mZP3 or GST: 100 μg (1 mg/mL) of antigen was mixed with Imject™ Alum Adjuvant (Thermo Fisher Scientific) at a volume ratio of 1:1, and three rounds of immunization schedule are performed by subcutaneous injection at 2-week intervals. The drug SS (Sigma-Aldrich) was dissolved in saline with indicated concentration (0.5 mg/kg) after referring to the previous studies (31, 37), then 100 μL of SS or the same volume of saline as the negative control were administered to mice *via* intraperitoneal injection once daily throughout the entire immunization program before the mating procedure. Finally, ten mice in each group were selected randomly for assessments of litter size followed by ovarian histological analysis, while the remaining fifteen mice in each group were used solely for ovarian tissue-related tests.

### Animal Mating and Litter Size Statistics

On day 36 after first administration, ten mice were coupled with normal males of similar age (one male for each female) with rotation of male mice conducted among the different cages daily. While females with successful mating were confirmed by monitoring of vaginal suppositories daily at 8:00 am during the mating period. Males were removed after coupling for 7 days. All males used in these experiments were previously confirmed to be fertile through mating with untreated females. The number of pups produced by each female were counted and compared with that of controls by 21–27 days after mating procedure.

### Sera Collection and Determination of the Antibody Levels

Serum samples were collected from mice on the day of mating then were stored at −80°C until use. The level of IgG in serum was determined by indirect enzyme-linked immunosorbent assay (iELISA). Briefly, 96-well plates (Nunc, Thermo Fisher Scientific) were coated with purified mZP3 or GST (400 ng/well) and incubated at 4°C overnight. Next, solutions in wells were replaced with blocking buffer (PBS solution containing 5% skimmed milk) then plates were incubated at room temperature (RT) for 1 hour. After washing wells once with PBS-T (PBS buffer containing 0.05% Tween 20), 100 μL of serial 2-fold dilutions of serum in blocking buffer (from 1:100 to 1:1638400) were added to triplicate wells followed by 1 hour incubation of plates at RT. Wells were then washed three times with PBS-T then 100 μL (0.2 μg/mL) of HRP-conjugated goat anti-mouse IgG (TransGen Biotech) diluted in blocking buffer was added per well. Next, binding between the secondary antibody and primary antibody was allowed to proceed for 1 hour at RT. After liquid was removed from wells, wells were washed three times with PBS-T then 100 μL 3,3′,5,5′-tetramethylbenzidine (TMB; TransGen Biotech) was added per well for 10 min to trigger colorimetric reaction. Finally, OD_450nm_ values were read using an Epoch-BioTek Microplate Reader after the reaction was terminated by adding of 3 M H_2_SO_4_ (50 μL/well). Determination of the antibody titer for each mouse was conducted based on whether average OD_450nm_ values of a given serum dilutions in triplicate (presented as the mean value plus three standard deviations) was greater than the mean value for the corresponding dilution of negative serum.

### Ovarian Collection and Histological Analysis

Statistical analysis of numbers of pups in each group was conducted on day 70 after the first immunization, then 10% chloral hydrate (Sigma-Aldrich) (10 mg/kg) was injected intraperitoneally into each mouse to anesthetize the animal then ovaries were removed for histological preparation and analysis of ovarian tissue section. Briefly, ovaries of ten mice in each group were fixed immediately by immersion into 4% paraformaldehyde (Beyotime Biotechnology) after collection, fixed tissues were maintained at 4°C for at least 48 hours then embedded in paraffin and sliced into 5 μm section, followed by hematoxylin and eosin (HE) staining steps with Hematoxylin and Eosin (HE) Staining Kit (Beyotime Biotechnology) according to the manufacturer's instructions. Ovaries tissues with HE staining were examined according to the method of Myers et al., to identify the mouse follicles at different developmental stages and accurately estimate the number by the number of granulosa cells, not morphology. Primary follicle is totally surrounded by a single layer of cubic granular cells, while mature follicles is surrounded by a part of cumulus cells (38).

### Evaluation of ROS Levels in Mouse Ovarian Cells

To assess ROS levels in mouse ovarian cells, a fluorescence probe-based method was used. First, five randomly selected mice in each group were euthanized to collect ovaries using the same method as mentioned above. Next, tissues were pulverized to generate single-celled suspensions by pressing the tissues into a 200-mesh sterile copper screen using a glass rod. Resulting cell suspensions were centrifuged at 300 × *g* for 10 min at 4°C then cell pellets were washed twice with Dulbecco's Modified Eagle's Medium (DMEM, Gibco, Carlsbad, CA, USA). Detection of ROS in ovarian cells was performed using a Reactive Oxygen Species Assay Kit (Beyotime Biotechnology) according to the manufacturer's instructions.

To further assess levels of OS-associated indicators in ovarian tissue, another five mice were randomly selected from each group for ovaries collection as well. Next, tissues were homogenized on ice at 1:10 (w/v) in saline (*PH* = 7.2) using a MagNA Lyser Tissue Homogenizer (Roche, Basel, Switzerland) then supernatants were obtained by centrifugation of homogenized specimens at 13,000 × *g* for 20 min at 4°C. Ovarian tissue supernatants were next evaluated for ROS and malondialdehyde (MDA) levels as well as total antioxidant capacity (T-AOC) using Mouse Reactive Oxygen Species ELISA Kit (MLBio, Shanghai, China), Malondialdehyde (MDA) Colorimetric Assay Kit (TBA method, Elabscience, Wuhan, China) and Total Antioxidant Capacity Colorimetric Assay Kit (Elabscience), respectively.

### Analysis of Mitochondrial Apoptosis-Related Proteins

Ovaries from the remaining five mice in each group were obtained using the same methods as described above then were frozen in liquid nitrogen. All ovarian samples in each group were pooled together and ground into a fine powder using a mortar and pestle. Next, total protein was extracted using a Tissue or Cell Total Protein Extraction Kit (Sangon Biotech, Shanghai, China) according to the manufacturer's instructions. Finally, protein concentrations were determined using a BCA Protein Assay Kit (Thermo Fisher Scientific) according to the manufacturer's instructions.

For Western blot analysis, protein samples were first separated by sodium dodecyl sulfate-polyacrylamide gel electrophoresis (SDS-PAGE) then proteins were electro-transferred to the methanol-activated PVDF membranes. Next, membranes were blocked then incubated with primary and secondary antibodies as described above for the iELISA; primary antibodies (Beyotime Biotechnology) included anti-Bax, anti-Bcl-2, anti-caspase-3 and anti-caspase-9 that were each diluted 1:1,000. After membranes were washed, they were developed based on a colorimetric reaction with ECL Western Blot Substrate (Solarbio) then developed blots were viewed using a ChemiDoc MP Image System (Bio-Rad). Differences in protein expression levels among different groups were analyzed using Image J software (Image J2x, Rawak Software Inc., Stuttgart, Germany).

### Statistical Analysis

All data are presented as the mean ± SD. A scatter diagram of antibody levels, litter sizes and correlations between litter size, as well as antibody titer or number of follicles were determined using nonparametric correlation (Spearman's correlation). Determinations of statistical significance of line charts and histograms associated data within each group were all performed using GraphPad Prism version 7.0 (GraphPad Software, San Diego, CA, USA) using either the Student's *t*-test for the comparison of two groups or one-way analysis of variance (ANOVA) for testing of more than two groups. A two-tailed *P* value <0.05 is considered to be statistically significant.

## Results

### Fertility Assessment of mZP3-Immunized Mice After SS Treatment

Statistical analysis of data obtained in this study revealed no differences in fertility rates among the three groups mZP3+SS, mZP3+Saline and GST+Saline, which were 90% (9/10), 70% (7/10) and 100% (10/10), respectively. However, values of average mean litter size per mouse in both mZP3+SS and mZP3+Saline groups were significantly lower at 4.3 (*P* < 0.05) and 2.4 (*P* < 0.01) than that of the GST+Saline negative group of 6.5 (Table 1). Notably, SS treatment of the mZP3+SS group led to higher average litter size without affecting the ZP3-induced antibody titers, which was ~29% higher than that of the drug control group mZP3+Saline, indicating a key role of OS in ZP3-vaccination-induced subfertility. These results suggest that mZP3-induced OS likely further caused subfertility on the basis of antibody-mediated fertility effects.

**Table 1:** Fertility of mice from different groups.

**Antigen**	**Mouse^a^**	**No. fertile**	**% Fertile^b^**	**Total^c^ pups**	**Mean litter size of mice^d^**	***P* ^e^**
mZP3 + SS	10	9	90	43	4.3 ± 2.41^*^	0.007
mZP3 + Saline	10	7	70	24	2.4 ± 1.43^**^	0.013
GST + Saline	10	10	100	65	6.5 ± 0.98	

a *Female BALB/C mice were immunized with mZP3 protein and control protein GST at 2 week intervals for three times, and treated with chemical drug SS and control saline daily though the whole immunization process before mating. All mice were subjected to mating on day 36 after the first immunization*.

b *Percentage of fertile mice were calculated by the fertile numbers divided by the total mated females in each group*.

c *Total number of pups of each group were counted 21~27 days after animals mating*.

d *Mean litter size of mice were calculated by the total pups divided by the total mated females in each group*.

e Significant differences (^*^P < 0.05 and ^**^P < 0.01) are presented between immunized groups and negative controls (GST+Saline). Values are the means ± SD.

**Figure 1:**
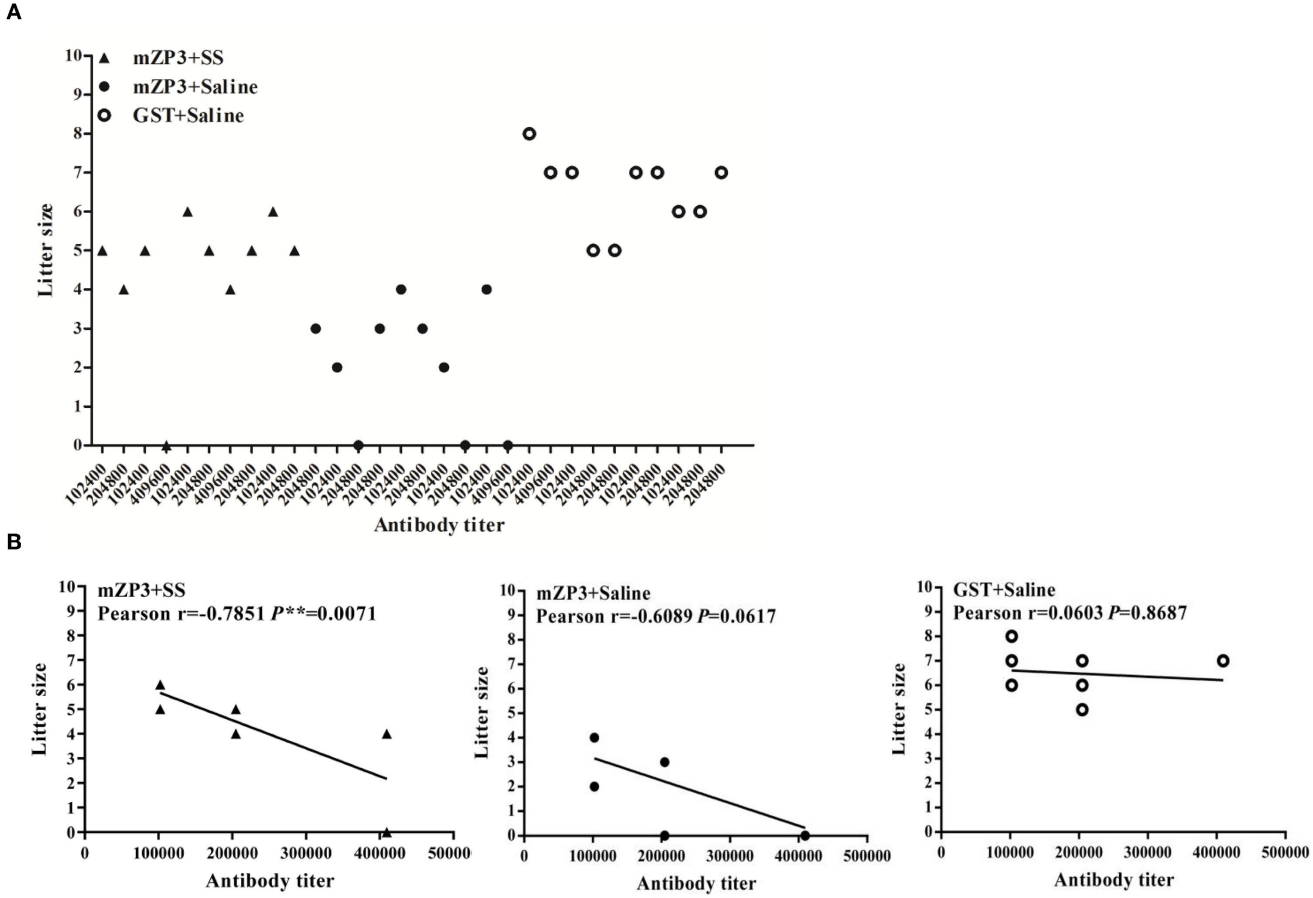
ZP3-specific antibody shows a significant negative correlation with litter size. **(A)** Scatter diagram of antibody levels and litter size after mating. The antibody levels of each mouse were determined by serum serial 2-fold dilution from 1:100 to 1:819200 in an iELISA assay, and corresponds to their own litter size. **(B)** Correlation analysis between antibody titer and litter size. The Pearson coefficient *r* value means the correlation between antibody titer and litter size, while *P* value <0.05 indicates credibility of *r* value.

Determination of antibody titer for each mouse in the different groups indicated that strong immune responses against mZP3 and GST were observed in all mice, including the infertile one (Figure 1A). GST group mice with anti-GST antibody titers that were equivalent to anti-mZP3 antibody titers of mZP3+SS and mZP3+Saline groups gave birth to the greatest numbers of pups, with no correlation found between anti-GST antibody titers and litter size (Pearson *r* = 0.0603). Moreover, no significant difference was observed in numbers of litters among individual mice in this group (*P* = 0.8687) (Figure 1B). Meanwhile, in the mZP3+SS group a negative correlation was found between antibody level and litter size that was significantly more negative (Pearson *r* = −0.7851) than that obtained for the Non-treated, mZP3-immunized group (Pearson *r* = −0.6089). Furthermore, the *P* values of mZP3+SS (*P* = 0.0071) and mZP3-immunized (*P* = 0.0617) groups both are <0.05, suggesting that the above correlation analysis is highly reliable (Figure 1B).

### SS-Administration Restored the OS Induced Follicle Depletion

**Figure 2:**
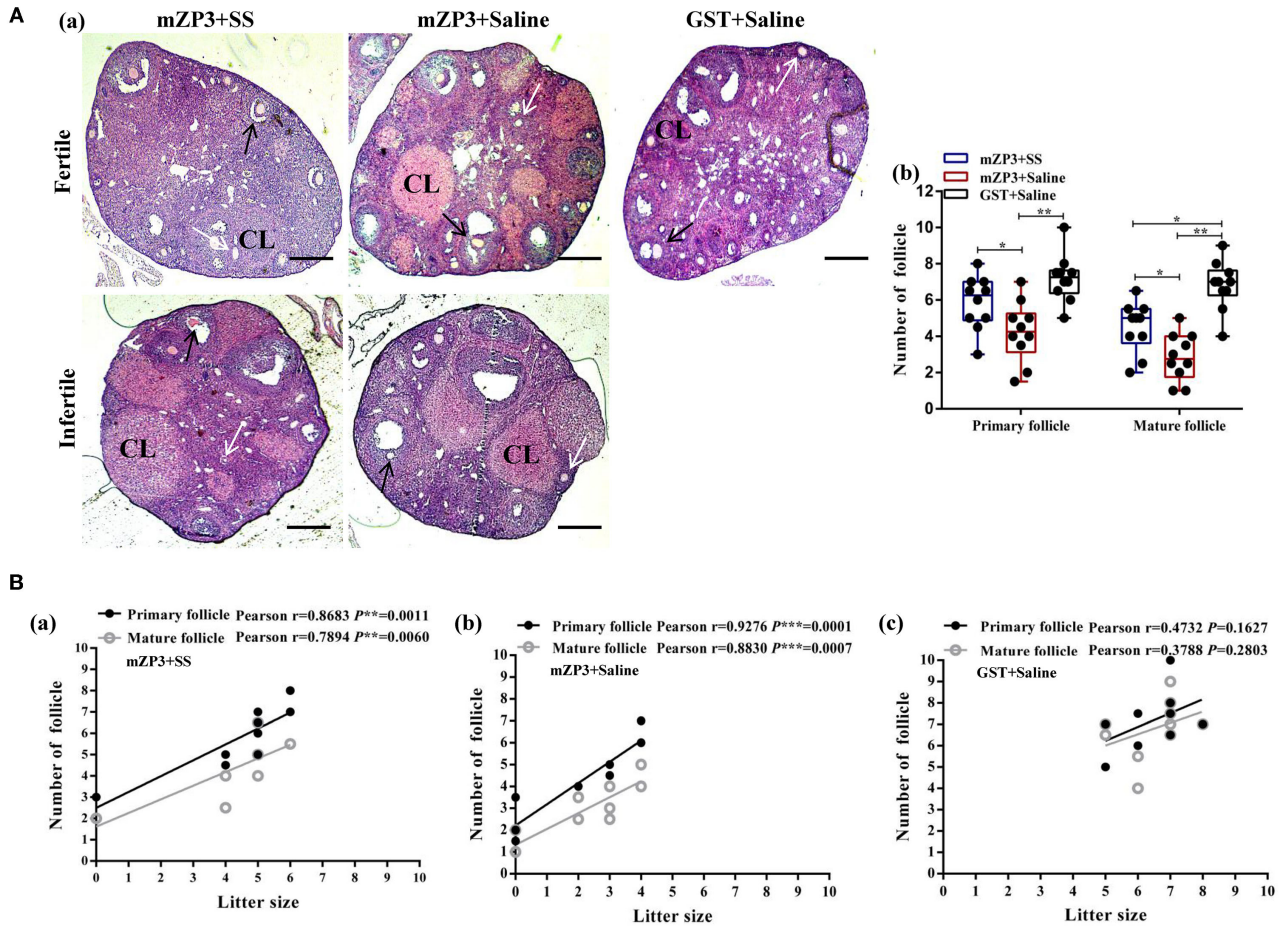
Follicle depletion is associated with litter size decline in mZP3 immunized mice. **(A)** Histological analysis of ovaries of mZP3+SS, mZP3+Saline and GST+Saline immunized mice. Mice ovaries were collected on day 70 after the first immunization, followed by tissue sectioning and HE staining for primary and mature follicles count (40×) (a), then performed statistical analysis among the indicated groups are marked as “*” or “**”, which denotes *P* < 0.05 and *P* < 0.01, respectively, and those with insignificant differences are not marked (*P* > 0.05) (b). CL: Corpus luteum. Black arrows indicate primary follicles; while arrows indicate mature follicles. **(B)** Correlation analysis between litter size and follicle number. The number of primary and mature follicles were counted based on the obtained ovarian sections above, and performed correlation analysis with litter size of mZP3+SS (a), mZP3+Saline (b) and GST+Saline (c) immunized mice. The Pearson coefficient *r* value means the correlation between antibody titer and litter size, while *P* value <0.05 indicates credibility of *r* value.

Notably, in this work a significant follicle number reduction, especially the mature one, were observed in ovaries of the mZP3+Saline group as compared to the negative control group. Specifically, mean numbers of primary and mature follicles dropped from 7.2 and 6.8, for the negative control group to 4.3 and 2.9, respectively, for the mZP3+Saline group (Figure 2Ab). Interestingly, numbers of corpus luteum (CL) increased significantly in both fertile and infertile mice (Figure 2Aa), but did not contribute to fertility, indicating that the follicles were abnormally disappeared. In addition, although numbers of follicles differed significantly among individual mice, the total number of follicles in mZP3-immunized mice were significantly increased in mice that received SS treatment, while primary follicle numbers were comparable to numbers for the negative control group (Figure 2Ab). More importantly, the correlation analysis revealed that numbers of primary and mature follicles were both significantly positively correlated with litter size (*r* = 0.8683 and *r* = 0.7894; *r* = 0.9276 and *r* = 0.8830) (Figure 2Ba,b). Surprisingly, the credibility of correlation analysis in the SS-treated, mZP3-immunized group (Figure 2Ba) decreased from *P* < 0.001 to *P* < 0.01 as compared to that of the untreated mZP3-immunized group (Figure 2Bb). Meanwhile, although not significant, the correlation between primary and mature follicles with litter size also decreased from Pearson *r* = 0.9276 and Pearson *r* = 0.8830, respectively (Figure 2Bb), in the mZP3+Saline group to Pearson *r* = 0.8683 and Pearson *r* = 0.7894 in mZP3+SS group, respectively (Figure 2Ba).

### OS Is Associated With ZP3-Induced Subfertility

**Figure 3:**
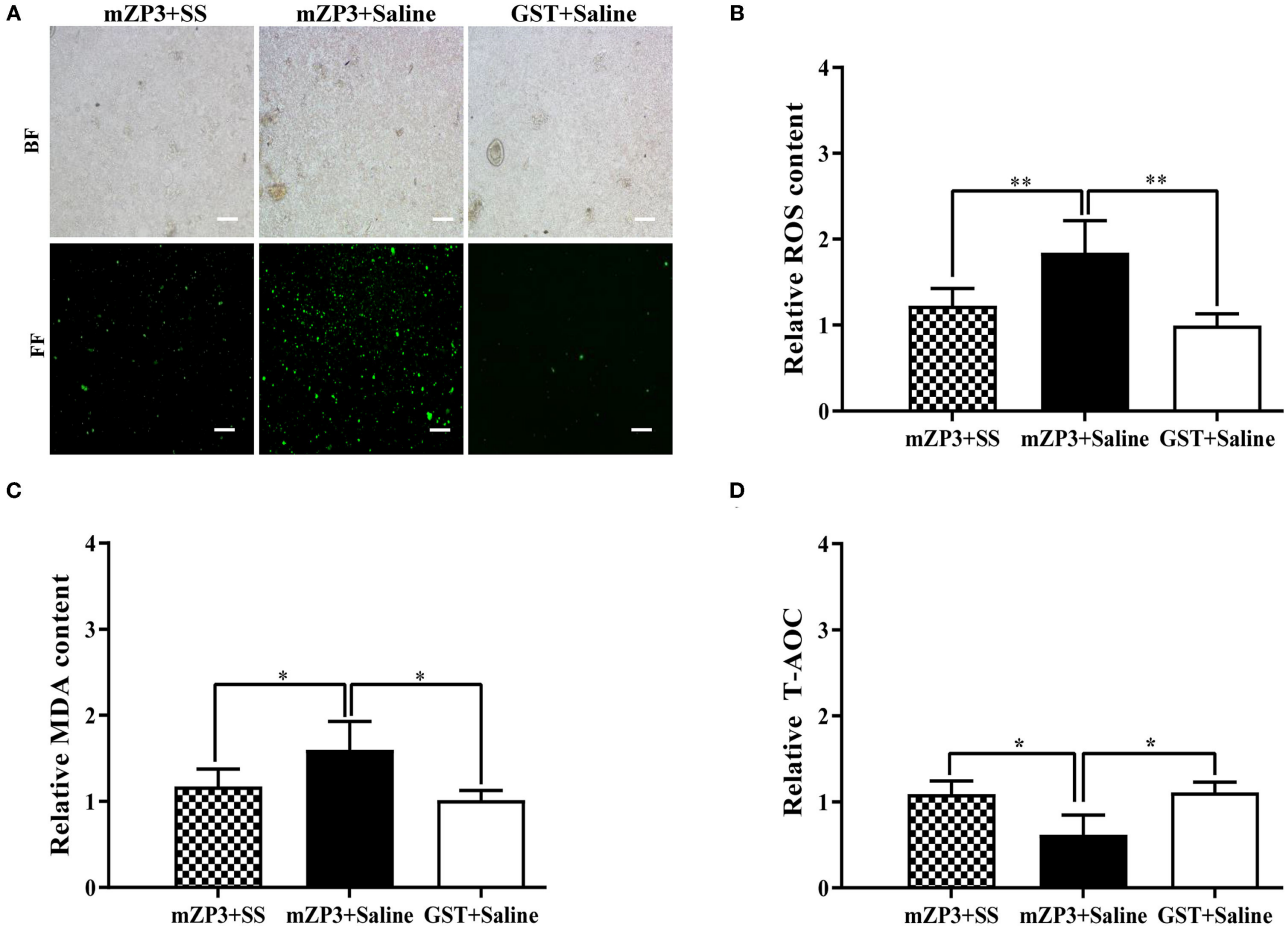
OS occurs in the ovarian cells of mZP3 immunized mice. Ovaries of all mice from different groups were collected on day 36 after the first immunization for the assessment of OS. **(A)** ROS content assay in the ovarian cells. The single ovarian cells of ovarian tissues were obtained after treatment with 200 mesh copper, and processed with evaluation of ROS content with fluorescent probe method and presented on bright field (BF) and fluorescent field (FF) (10×). **(B)** ROS content assay in the ovarian tissue. The ovarian tissues were subjected to homogenization, then the supernatant were obtained for ROS test. Statistical significance between indicated groups are marked as “**”, which means *P* < 0.01. **(C)** Accumulation of malondialdehyde (MDA) in the ovarian cells. The ovarian tissue supernatant were harvested same as panel B, and conducted to detection of MDA. Statistical significance between indicated groups are marked as “*”, which means *P* < 0.05. **(D)** T-AOC of ovarian cells reduced in mZP3 immunized mice. The ovarian tissue supernatant were harvested same as panel B, and conducted to detection of ROS. Statistical significance between indicated groups are marked as “*”, which means *P* < 0.05, and those with insignificant differences are not marked (*P* > 0.05).

Due to the abovementioned beneficial effects of SS treatment for improving fertility and follicle development, evaluations of ovarian OS levels were conducted concurrently to reveal the mechanism underlying development of subfertility in mZP3-immunized mice. As shown in Figure 3A, obvious OS accumulation in ovarian cells was observed in mZP3-immunized mice. Subsequently, this accumulation was further confirmed by checking ovarian tissue homogenate ROS levels, and declared that the ROS level was elevated in ovarian tissue by about 84% as compared to that of the negative control group (Figure 3B). Additionally, MDA content associated with membrane damage also showed a significant increase (by about 58%), as expected (Figure 3C). In addition, evaluation of ovarian T-AOC demonstrated a significant decrease also when abovementioned metabolic ROS homeostasis was disrupted, with consistent results observed here whereby T-AOC dropped by about 40% (Figure 3D). Although mZP3-induced ovarian OS was not completely diminished by SS treatment, administration of SS during mZP3 immunization alleviated OS to some degree, such that levels of all OS-related indicators in mZP3+SS mice were more than 40% lower than corresponding indicators for mice immunized with mZP3 alone.

### Vaccination-Induced OS Promotes Apoptosis of Ovarian Cells

**Figure 4:**
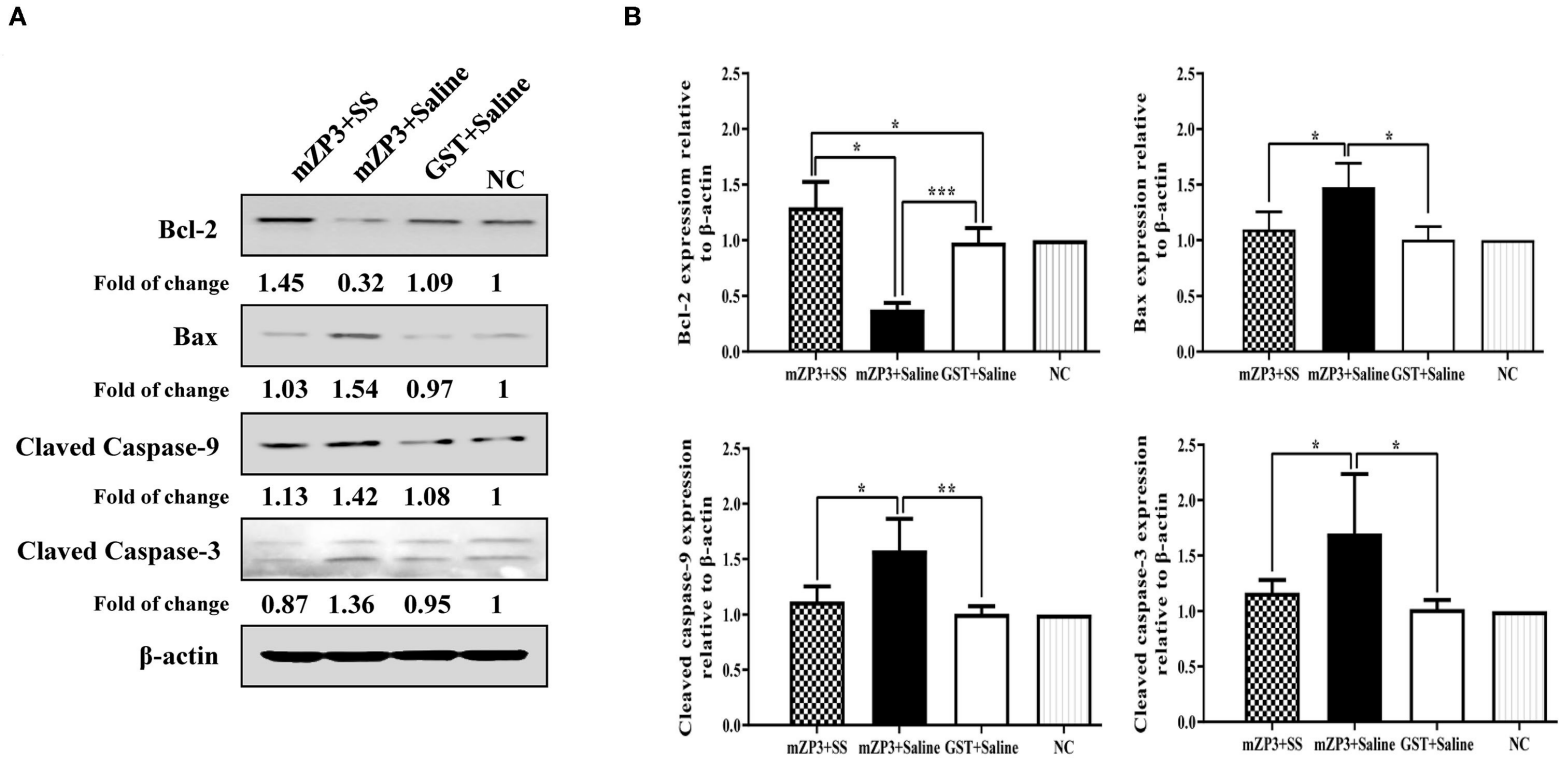
The mZP3 vaccination causes apoptosis of ovarian cells in mice. **(A)** Apoptosis-related proteins increases in ovarian cells of mZP3 immunized mice. Ovaries were collected on day 36 after the first immunization, then performed the extraction of total protein and Western blot for detection of apoptosis-related proteins. Mock-immunized and treated mice were used as the negative control (NC). **(B)** Statistical analysis of Bax, Bcl-2, cleaved-caspase-9 and cleaved-caspase-3 of panel a. All data comes from the three biological replicates of each group. Statistical significance between indicated groups are marked as “*”, “**” or “***”, which denotes *P* < 0.05, *P* < 0.01 and *P* < 0.001, respectively, and those with insignificant differences are not marked (*P* > 0.05).

Considering that numbers of ovarian follicles and ROS homeostasis both declined in mZP3-immunized mice, the hypothesis that ovarian pathology leads to mitochondrial apoptosis and abnormal follicular development may be correct. To further test this hypothesis, activation of key apoptosis-related proteins caspase-3 and caspase-9 as well as changes in expression levels of pro-apoptotic protein Bax and anti-apoptotic protein Bcl-2 were assessed to determine the degree of cell apoptosis in the ovary. Toward this goal, Western blot results demonstrated that the first three aforementioned proteins were all up-regulated after mZP3 immunization, while expression of Bcl-2 protein was significantly reduced (Figure 4A). Interestingly, the mZP3 effect for promoting apoptosis was significantly reversed by simultaneous treatment of SS. Specifically, relative levels of activated caspase-3 and caspase-9 in the mZP3+SS group only increased by 17 and 12%, respectively, as compared to corresponding levels in GST-immunized mice and mock-treated mice, while corresponding levels in mZP3-immunized mice were increased by ~70% and ~58%, respectively (Figure 4B). Meanwhile, up-regulation of Bax protein expression in mZP3-immunized, SS-treated mice was reduced by 30% as compared to that of untreated mZP3-immunized mice. In addition, it is worth noting that SS treatment not only restored Bcl-2 expression levels in mZP3-immunized mice, but led to elevated levels as compared to those of untreated, but immunized mice. In the final analysis, levels of anti-apoptosis indicators in ovarian cells of mZP3-immunized mice treated with the OS-specific drug SS were all significantly greater (*P* < 0.05) than corresponding levels observed for untreated, mZP3-immunized mice based on the detection of apoptosis-associated proteins (Figure 4B).

## Discussion

By targeting to block sperm-preferred receptors or enhancing antibody-mediated steric hindrance of sperm, high-titer antibodies produced against ZP3 have been shown to inhibit fertilization, which is a key mechanism of infertility (2, 3, 39). Moreover, the presence of auto-antibodies to ZP in sera of infertile women has also been shown to be closely related to infertility (40), and negatively correlated with fertilization *in vitro* and fertility *in vivo* (6, 41). Although no evidence exists to declared that the presence of ZP3 antibodies affects the formation of the follicular ZP structure, and once this structure was formed thus ZP3 antibodies had minimum effect on development of the fertilized egg after implantation (42), the fundamental role of ZP3-specific antibodies within the vaccination induced infertility is confirmed by present study. Our data suggested that mice immunized with recombinant mZP3 protein containing intact pathogenic epitopes did indeed exhibit ovarian pathological damage, whereby mZP3-immunized mice had higher titers of ZP3-specific antibodies gave birth to fewer pups. In addition, correlation analysis revealed a significant negative correlation between antibody level and litter size, indicating the leading role of ZP3-specific antibody-mediated subfertility. More importantly, once the progressive declining of follicles was interrupted by SS treatment, the coefficient *r* value representing the correlation between antibody and litter size has changed from 0.0617 to 0.0071, of which further confirm the conclusion that antibodies dominate infertility.

Here, results obtained *via* examination of ovarian tissue sections of fertile and infertile mice suggested that numbers of follicles, especially mature follicles, was significantly reduced and associated with fewer litters. Furthermore, highly significant differences were observed in ovarian follicles numbers of mice belonging to different experimental groups, in spite of the fact that mice with near equivalent antibody titer to their mZP3. Although the specific underlying mechanism for this effect is unknown, these results strongly indicated closely relationship between ZP3 administration and follicle number decline of immunized mice as reported previously (8). Nevertheless, it is speculated that AOD caused by T cells recognizing a pathogenic T cell epitope within the ZP3 protein accelerates depletion of follicles *via* an unknown process (4, 43). Moreover, the CL is the collapsed structure of the follicular wall remaining in the ovary after ovulation, is closely related to the ovulation cycle of the female individual (44, 45), and both increased significantly in the SS-treated and ZP3-immunized mice without SS treatment, especially the infertile one. However, neither the number of follicles nor the litter size was improved in those mice with increased CL and showed no relationship, indicating an unknown factor of follicles disappearance caused subfertility. Meanwhile, considering ROS resulted inflammation and tissue injury (13, 19), the AOD induced by ZP3 immunization has been shown to possess characteristics of inflammation, follicle dysplasia and follicle failure, etc. (4, 5, 46), prompting us to further assess ovarian ROS levels. As expected, ROS accumulation in ovarian cells and whole tissues reached high levels, while MDA and T-AOC levels were increased and decreased, respectively, suggesting a serious OS and potential mitochondrial dysfunction in ovarian cells of ZP3-immunized mice, and the resulting side effects maybe also associated with the inflammation in the AOD model (11, 18, 19). Moreover, there are conclusive evidence has demonstrated that mitochondrial OS is a key activator of apoptosis-induced follicles depletion (47–50). Therefore, evaluation of caspase, anti-apoptotic and pro-apoptotic protein levels was further examined and it appeared that mitochondrial apoptotic pathway was involved in the progression of AOD, with apoptosis-induced follicular damage found to directly cause ovarian inflammation-associated reduction of follicle number. Meanwhile, abnormal mitochondria metabolism caused OS is probably involved in the apoptosis of ovarian cells and thus affect the development of follicles, which may provide an interpretation for the inconsistency of causality among increased CL and follicular reduction or litter decline after ZP3 vaccination.

Previously study demonstrated that SS treatment was shown to significantly promote development of follicles, oocytes and embryos *in vitro* by reducing ROS and increasing T-AOC levels (32). Meanwhile, SS treatment has also been shown to significantly increase OS levels in ovaries and uterus by inhibiting expression of activated caspase protein (31). Here, SS treatment of mZP3-immunized mice alleviated ovarian dysfunction by enhancing anti-OS ability and decreasing apoptosis levels. Statistical analysis of ROS and MDA results indicated levels of both were significantly decreased and that T-AOC level was significantly increased as compared to corresponding levels in untreated, mZP3-immunized mice. Therefore, our results suggested that OS depleted follicles *via* apoptosis, as reported previously (51–53). Moreover, as is a lysosome-mediated degradation process for Non-essential or damaged cell components, autophagy may participates in the above abovementioned OS-induced mitochondrial dysfunction of mZP3-immunized mice ovaries, but more investigations is needed (54). It is worth noting that our results demonstrated that SS co-administration with mZP3 immunization not only restored the number of follicles, but also significantly promoted increased litter sizes for almost all SS-treated, mZP3-immunized mice (*P* < 0.05); by contrast, an inverse correlation was found between litter size and anti-mZP3 antibody titers (*P* < 0.01). Additional research by Said et al., has also found that SS-treatment maintained serum levels of follicle-stimulating hormone (FSH) and estradiol (E2) to prevent permanent follicle loss and further stimulate nuclear antigen (PCNA)-mediated follicular proliferation that ultimately improved ovarian function (31). Thus, these results indicate that the infertility mechanism caused by ZP3 vaccination induced ovarian dysfunction is extremely complicated, while the role of OS-related interlocking events during follicular development also should not be underestimated. In fact, during later stages of follicular development, reduction of follicle numbers in mZP3-immunized mice is likely the fundamental cause of follicle exhaustion that is associated with infertility (55, 56).

## Conclusion

Results of the present study reveal a vital role of OS-induced follicle loss for the first time in the ZP3-based immunocontraception model. Specifically, the normal metabolism of mitochondria in ovarian cells was disrupt by the OS caused by inflammation, whereby the accumulation of ROS triggers the process of apoptosis and follicles depletion, and ultimately leading to the fertility decline of fertility. However, co-administration of SS with ZP3 immunization not only reversed the above harmful process, but also improved follicle number and litter size either effectively. Taken together, these results indicated that OS and related side effects are responsible for observed follicle loss and associated with closely subfertility. Thus, infertility model resulted from the dominant effect of ZP3-specific antibody-mediated infertility during early stages of immunization and a Non-dominant role of OS at later stages of follicle development together.

## Data Availability Statement

The original contributions presented in the study are included in the article/supplementary material, further inquiries can be directed to the corresponding author.

## Ethics Statement

The animal study was reviewed and approved by Committee on Ethical Use of Animals of Northwest A&F University.

## Author Contributions

YN and E-MZ: conceived and designed the experiments. BZ and GQ: performed the experiments. BZ and YN: analyzed the data. BZ: writing-original draft preparation. YN: writing-reviewing and editing. All authors contributed to the article and approved the submitted version.

## Funding

This work was supported by the grants from the National Natural Science Foundation of China awarded to YN (No. 31672534).

## Conflict of Interest

The authors declare that the research was conducted in the absence of any commercial or financial relationships that could be construed as a potential conflict of interest.

## Publisher's Note

All claims expressed in this article are solely those of the authors and do not necessarily represent those of their affiliated organizations, or those of the publisher, the editors and the reviewers. Any product that may be evaluated in this article, or claim that may be made by its manufacturer, is not guaranteed or endorsed by the publisher.
